# Adrenergic pathways in glycine-mediated feeding behavior: Evidence from layer chickens

**DOI:** 10.1016/j.psj.2025.105338

**Published:** 2025-05-26

**Authors:** Hamed Zarei, Fatemeh Kashefi, Keyvan Hasani

**Affiliations:** a Department of Biology, CT.C., Islamic Azad University, Tehran, Iran; b Department of Basic Sciences of Veterinary Medicine, Garmsar Branch, Islamic Azad University, Garmsar, Iran

**Keywords:** Glycine, Adrenergic receptors, Food intake, Intracerebroventricular injection, Layer-type chickens

## Abstract

Glycinergic and adrenergic systems are integral to the regulation of meal consumption in avian species; however, the interactions between these systems have not been previously documented. This investigation was conducted to explore the interplay between the central glycinergic and adrenergic systems in regulating feed consumption among egg-type chickens. To this end, six separate experiments were performed. The first experiment involved intracerebroventricular (ICV) infusion of normal saline and glycine at different doses (50, 100, and 200 nmol) to determine its effective dose. In experiments 2-6, sub-effective doses of various adrenergic receptor antagonists—prazosin (an antagonist of α1 receptor), yohimbine (an antagonist of α2 receptor), metoprolol (an antagonist of β1 receptor), ICI 118.551 (an antagonist of β2 receptor), and SR 59230R (an antagonist of β3 receptor)—were co-administered with an effective dose of glycine to assess the potential involvement of adrenergic receptors in glycine-mediated feed intake. The cumulative feed consumption was subsequently measured at intervals of 30, 60, and 120 minutes post-administration. The findings indicated that glycine infusion at doses of 100 and 200 nmol significantly reduced meal consumption in layer chickens compared to the saline-treated control group (*P* < 0.05). Additionally, co-infusion of a β2 receptor antagonist attenuated the hypophagic effects typically induced by glycine (*P* < 0.05). However, co-administration of α1, α2, β1, and β3 receptor antagonists did not alter glycine-induced hypophagia (*P* ≥ 0.05). These findings suggest that the role of glycine in decreasing feed consumption in layer chickens could be facilitated by β2 adrenergic receptors**.**

## Graphical abstract

**Figure:**
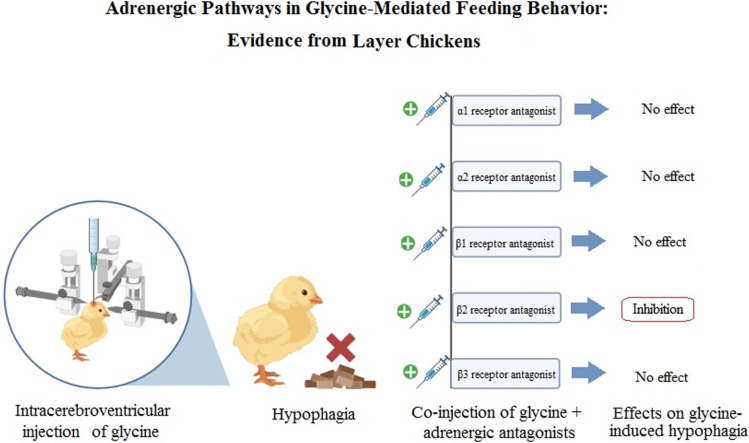

## Introduction

Appetite regulation is a critical physiological mechanism responsible for controlling survival and welfare in animals and humans. Optimal appetite regulation will enable proper consumption of sufficient nutrients to maintain metabolic requirements but will not allow for overconsumption and obesity and its concomitant diseases (Hwalla and Jaafar, 2020). Understanding the various regulators involved in appetite control, along with their interrelationships, is crucial for the effective manipulation of feeding behavior (Watts et al., 2022). This is particularly significant in avian species, where different bird species exhibit different consumption patterns and responses to dietary changes (Ríos et al., 2014).

Glycine is a multifaceted molecule that serves as both a nonessential amino acid and a neurotransmitter within the vertebrate central nervous system (CNS) (Salceda, 2022). It is detectable in various brain regions, including the amygdala, prefrontal cortex, hippocampus, cerebellum, hypothalamus, ventral tegmental area (VTA), nucleus accumbens (NAc), substantia nigra (SN), brain stem, and spinal cord (He and Wu, 2020). Glycine's inhibitory action is primarily mediated through its binding to ligand-gated chloride channels, which hyperpolarize postsynaptic neurons, thereby increasing the threshold required to stimulate neurons (Pressey et al., 2023). Glycine also interacts with N-methyl-D-aspartate (NMDA) receptors, which are cation-selective and excitatory, suggesting a potential dual role in synaptic plasticity and appetite regulation via glutamatergic pathways (Michalski and Furukawa, 2024). In the context of food intake regulation, glycine has been identified as a significant component. Research in broiler chickens has shown that glycine can decrease meal consumption, an effect that is enhanced by dopamine (DA) through D1 receptors (Rahimi et al., 2021a). This interaction suggests a complex interplay between glycine and DA in modulating feeding behavior. Furthermore, the hypophagic effect of glycine in chickens is attributed to its release from nerve terminals and subsequent binding to strychnine-sensitive glycine receptors, leading to postsynaptic neuronal hyperpolarization (Rahimi et al., 2021b). The importance of dietary glycine in early nutrition for chickens has been noted, emphasizing its role as a limiting factor in nutritional development (Lee et al., 2022; Hilliar et al., 2019). Overall, glycine's dual function as a neurotransmitter and amino acid positions it as a critical regulator of neural communication and feeding behavior across different species.

In addition to glycine, the adrenergic system, which interacts with catecholamines like norepinephrine (NE) and epinephrine (E), plays a pivotal role in modulating appetite (Mahdavi et al., 2024). Adrenergic receptors are distributed throughout the CNS, particularly in areas like the hypothalamus and brainstem that control feeding behavior (Pruccoli et al., 2024). These receptors are categorized into alpha (α) and beta (β) types, with subtypes α1, α2, β1, β2, and β3 (Ciccarelli et al., 2013). Alpha adrenergic receptors, particularly α2, have been shown to stimulate meal consumption. For example, infusion of clonidine (an α2 receptor agonist) or NE into the paraventricular nucleus (PVN) stimulates feeding behavior in rats and domestic fowl (Denbow and Sheppard, 1993; Leibowitz et al., 1984; Shor-Posner et al., 1988). This effect is blocked by yohimbine (an antagonist of α2 receptor) but not prazosin (an antagonist of α1 receptor) (Zendehdel and Hassanpour, 2014). However, species-specific differences exist: while α2 receptor activation increases meal consumption in chickens (meat-type) (Bungo et al., 1999), conflicting results have been reported for layer-type chickens, where NE administration may not significantly affect feeding (Denbow et al., 1981). Beta receptors generally exhibit anorexigenic effects by suppressing food intake. Central infusion of salbutamol (a β2 receptor agonist) decreases nutritional consumption in rats, while ICV injection of BRL37344 (a β3 receptor agonist) significantly reduces feed consumption in rats and chickens (Kanzler et al., 2011). In meat-type chickens, ICV injection of isoproterenol (a nonselective β1 and β2 receptor agonist) decreases both food and water intake (Baghbanzadeh et al., 2010). Adrenergic activity also interacts with other factors such as leptin (in broilers) (Zendehdel et al., 2020), serotonin (in layer-type chickens) (Zendehdel et al., 2017b), ghrelin (in broilers) (Zendehdel and Hassanpour, 2014), nociceptin/orphanin FQ (in broilers) (Zendehdel et al., 2017a), DA (in layer-type chickens) (Zanganeh et al., 2021), histamine (in broilers) (Mirnaghizadeh et al., 2017), and nesfatin-1 (in broilers) (Ashtari-Tavandashti et al., 2022) in regulating food intake. This interplay suggests a complex regulatory mechanism for feeding behavior that involves multiple pathways and systems.

Recent investigations have begun to illuminate the connections between glycine and the adrenergic system in the context of various physiological functions. These studies emphasize the anatomical intersections between glycinergic and adrenergic circuits within brain regions crucial for appetite control (Tohyama et al., 1989). A study using high-performance liquid chromatography (HPLC) also showed that glycine induced 22 % less catecholamine release compared to acetylcholine. On the other hand, the NE-to-E ratio differed significantly: acetylcholine stimulation yielded a ratio of 1.6, favoring NE release, while glycine stimulation resulted in a ratio of 0.6, indicating a preference for E secretion (Yadid et al., 1992). On the other hand, it has been shown that glycine can completely counteract the inhibitory effects of Kynurenic acid (KYN) on NMDA-induced NE release (Ransom and Deschenes, 1988). Although no research has yet examined the interactions between these two systems specifically in relation to food intake, the findings accumulated thus far indicate a plausible connection between these systems within the neurobiological mechanisms that regulate feeding behavior. Therefore, our research aims to delve deeper into the specific functions of adrenergic pathways in glycine-regulated feeding behavior in neonatal layer chicks. By examining these interactions across different avian species, we aspire to generate valuable information that will contribute to both fundamental knowledge and practical applications in poultry management practices.

## Materials and methods

### Procurement of animals

Female Hy-Line chicks (one-day-old) were sourced from Simorgh Co., a prominent egg production firm based in Iran. These chicks were initially housed collectively under consistent lighting conditions at a controlled temperature of 31 ± 2°C for an initial period of two days. Following this acclimation period, they were randomly assigned to individual boxes. Throughout the experimental phase, the chicks had unlimited access to a starter feed formulated to deliver 21 % protein and 2850 kcal/kg of metabolizable energy, which was specifically designed to promote the growth and development of young chickens (Table 1). At five days of age, the chicks received pharmacological treatments via injections (Eslam-Aghdam et al., 2024). To maintain uniform conditions before ICV infusion, the chicks were subjected to a three-hour fasting period, although they had continuous access to water during the study (Mahdavi et al., 2023). All procedures adhered to the animal care regulations established in Iran and the guidelines provided by the National Institutes of Health (USA) for the care and use of laboratory animals. The Animal Ethics Committee of the Central Tehran Branch of Islamic Azad University, Tehran, Iran (IR.IAU.CTB.REC.1403.187), granted approval for all experimental procedures.

**Table 1:** Ingredient and nutrient analysis of experimental diet.

Ingredient (%)		Nutrient analysis	
Corn	52.85	ME, kcal/g	2850
Soybean meal, 48 % CP	31.57	Crude protein (%)	21
Wheat	5	Linoleic acid (%)	1.69
Gluten meal, 61 % CP	2.50	Crude fiber (%)	3.55
Wheat bran	2.47	Calcium (%)	1
Di-calcium phosphate	1.92	Available phosphorus (%)	0. 5
Oyster shell	1.23	Sodium (%)	0.15
Soybean oil	1.00	Potassium (%)	0.96
Mineral premix	0.25	Chlorine (%)	0.17
Vitamin premix	0.25	Choline (%)	1.30
Sodium bicarbonate	0.21	Arginine (%)	1.14
Sodium chloride	0.20	Isoleucine (%)	0.73
Acidifier	0.15	Lysine (%)	1.21
DL-Methionine	0.10	Methionine (%)	0.49
Toxin binder	0.10	Methionine + cystine (%)	0.83
L-Lysine HCl	0.05	Threonine (%)	0.70
Vitamin D_3_	0.1	Tryptophan (%)	0.20
Multi enzyme	0.05	Valine (%)	0.78

Diet composition totals 100 %.

ME: metabolisable energy, CP: crude protein, per kg of diet, the mineral supplement contains 35.2 g manganese from MnSO4∙H2O; 22 g iron from FeSO4∙H2O; 35.2 g zinc from ZnO; 4.4 g copper from CuSO4∙5H2O; 0.68 g iodine from ethylene diamine dihydroiodide; 0.12 g seleniumfrom Na2SeO3. The vitamin supplement contains 1.188 g of retinyl acetate, 0.033 g of dl-α-tocopheryl acetate, 8.84 g of tocopherol, 1.32 g of menadione, 0.88 g of thiamine, 2.64 g of riboflavin, 13.2 g of nicotinic acid, 4.4 g of pantothenic acid, 1.76 g of pyridoxin, 0.022 g of biotin, 0.36 g of folic acid, 1500 mg of choline chloride.

### Drugs

The pharmacological substances used in the present study were obtained from Sigma Co. (USA) and included glycine, prazosin (an α1 adrenergic blocker at 10 nmol), yohimbine (an α2 adrenergic blocker at 13 nmol), metoprolol (a β1 adrenergic blocker at 24 nmol), ICI 118.551 (a β2 adrenergic blocker at 5 nmol), and SR 59230R (a β3 adrenergic blocker at 20 nmol). These agents were initially dissolved in absolute dimethyl sulfoxide (DMSO) and subsequently diluted with a 0.85 % saline solution containing Evans blue, adhering to a dilution ratio of 1:250, which resulted in a final DMSO concentration of 0.4 %. Importantly, this concentration of DMSO has been shown to be noncytotoxic (Hakura et al., 2010). For the control treatment, a solution of normal saline mixed with DMSO (0.4 %) and Evans blue was used.

### Injections and food intake measurement

The experimental procedure commenced with weighing the chicks, followed by ICV infusions performed via a Hamilton microsyringe (Switzerland) without the use of anesthesia. This procedure builds upon techniques developed by Tachibana et al. (2022) and uses a specialized apparatus to position the chick's head at a precise 45-degree angle. This arrangement guarantees that the calvarium is correctly positioned in relation to the working surface. A small opening was made in a transparent plate, which was subsequently placed over the right lateral ventricle of each chick. A syringe was carefully inserted 4 mm into the ventricle through this aperture, with the aim of minimizing physiological stress during the injection procedure (Davis et al., 1979). A total volume of 10 µL per chick was administered for all treatments, including control and experimental groups, with each drug diluted to its target dose in this fixed volume. The pharmacological substances were injected in succession, as outlined in Table 2, with dosages determined from previous research to guarantee both effectiveness and safety (Ashtari-Tavandashti et al., 2022; Rahimi et al., 2021a). Once the injections were finished, the chicks were placed back into their respective boxes, where they could access water and food without any restrictions. To assess feed consumption accurately, measurements were taken at 30, 60, and 120 minutes after administration. Feed consumption was calculated relative to body weight to account for differences in body mass and eliminate potential confounding variables. Each chick underwent only one experimental trial to avoid any behavioral biases. The precision of the injection sites was confirmed by analyzing frozen brain tissue slices for the presence of Evans blue dye, ensuring that only data from layer-type chickens with verified infusion sites were included in the final analysis.

**Table 2:** Injection procedurefor experiments 1 through 6.

Exp. 1	Injections
Treatments	
I	CS*
II	Glycine (50 nmol)
III	Glycine (100 nmol)
IV	Glycine (200 nmol)

Exp. 2	Injections
Treatments	
I	CS *
II	parazosin (10 nmol)
III	Glycine (200 nmol)
IV	parazosin (10 nmol) + Glycine (200 nmol)

Exp. 3	Injections
Treatments	
I	CS *
II	yohimbine (13 nmol)
III	Glycine (200 nmol)
IV	yohimbine (13 nmol) + Glycine (200 nmol))

Exp. 4	Injections
Treatments	
I	CS *
II	metoprolol (24 nmol)
III	Glycine (200 nmol)
IV	metoprolol (24 nmol) + Glycine (200 nmol)

Exp. 5	Injections
Treatments	
I	CS *
II	ICI 118,551 (5 nmol)
III	Glycine (200 nmol)
IV	ICI 118,551 (5 nmol)+ Glycine (200 nmol)

Exp. 6	Injections
Treatments	
I	CS *
II	SR 59230R (20 nmol)
III	Glycine (200 nmol)
IV	SR 59230R (20 nmol) + Glycine (200 nmol)

CS: control solution (normal saline +0.4 % DMSO + 0.1 % Evans Blue), parazosin: α_1_ receptor antagonist, yohimbine: α_2_ receptor antagonist, metoprolol: β_1_ adrenergic receptor antagonist, ICI 118,551: β_2_ adrenergic receptor antagonist, SR 59230R: β_3_ adrenergic receptor antagonist).

*n* = 11 chicken per group.

### Statistical analysis

Two-way repeated measures analysis of variance (ANOVA) was conducted to assess cumulative nutritional intake normalized to body weight. To compare means across different groups, the Tukey‒Kramer post hoc test was applied, with a significance level established at *P* < 0.05. All findings are reported as the mean values with the standard error of the mean (SEM) included.

## Results

### Determination of the effective dose of glycine

**Fig. 1:**
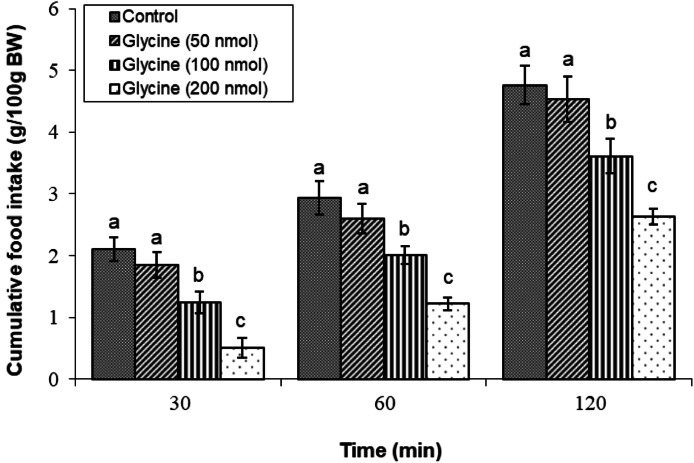
Changes in cumulative feed intake following ICV injection of glycine (50, 100 and 200 nmol) in young layer-type chickens (*n* = 11/group). The findings are shown as the mean ± SEM. Significant differences between treatment groups are marked with distinct letters (a, b, and c) at a P-value of less than 0.05. [Time impact: F(3,40) = 1819.10, *P* < 0.05; treatment impact: F(2,40) = 3990.07, *P* < 0.05; treatment × time interaction: F(6,80) = 41.01; *P* < 0.05].

The initial experiment aimed to establish the effective dose of glycine that induces hypophagia in neonatal layer chickens. Following the infusion of various doses, doses of 100, and 200 nmol of glycine significantly decreased feed consumption compared to control treatment (*P* < 0.05) (Fig. 1).

### Effects of adrenergic antagonists on glycine-induced hypophagia

#### Parazosin (an α1 receptor antagonist)

**Fig. 2:**
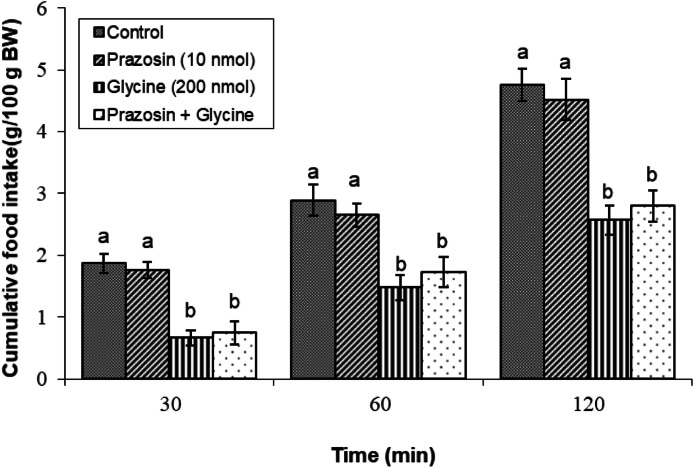
Changes in cumulative feed intake following ICV injection of parazosin (10 nmol), glycine (200 nmol) or their combination in young layer-type chickens (*n* = 11/group). parazosin: α1 receptor antagonist. The findings are shown as the mean ± SEM. Significant differences between treatment groups are marked with distinct letters (a and b) at a P-value of less than 0.05. [Time impact: F(3,40) = 2125.63, *P* < 0.05; treatment impact: F(2,40) = 4138.85, *P* < 0.05; treatment × time interaction: F(6,80) = 30.18; *P* < 0.05].

When compared to a glycine-only mixture, adding parazosin (10 nmol) to glycine (200 nmol) did not have a significant impact on glycine-induced hypophagia (*P* ≥ 0.05). This indicates that α1 adrenergic receptors likely do not play a significant role in modulating the hypophagic effect of glycine (Fig. 2).

#### Yohimbine (an α2 adrenergic receptor antagonist)

**Fig. 3:**
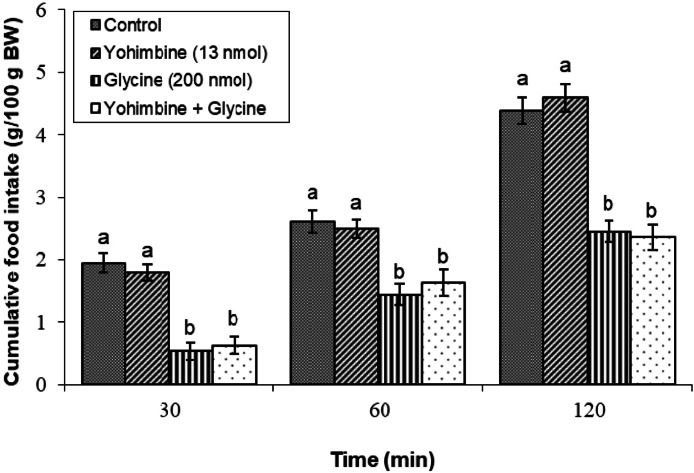
Changes in cumulative feed intake following ICV injection of yohimbine (13 nmol), glycine (200 nmol) or their combination in young layer-type chickens (*n* = 11/group). Yohimbine: an α2 receptor antagonist. The findings are shown as the mean ± SEM. Significant differences between treatment groups are marked with distinct letters (a and b) at a P-value of less than 0.05. [Time impact: F(3,40) = 2871.62, *P* < 0.05; treatment impact: F(2,40) = 3176.61, *P* < 0.05; treatment × time interaction: F(6,80) = 29.98; *P* < 0.05].

The combination of yohimbine (13 nmol) with glycine did not alter feeding behavior compared to the group receiving glycine alone (*P* ≥ 0.05), indicating a limited and non-significant involvement of α2 adrenergic receptors in this context (Fig. 3).

#### Metoprolol (β1 adrenergic receptor antagonist)

**Fig. 4:**
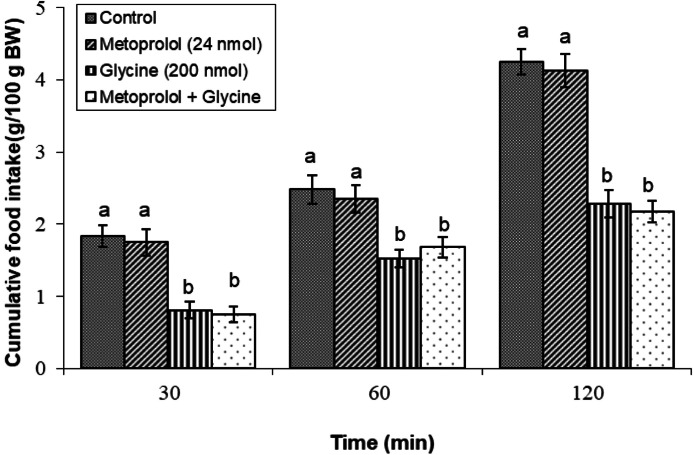
Changes in cumulative feed intake following ICV injection of metoprolol (24 nmol), glycine (200 nmol) or their combination in young layer-type chickens (*n* = 11/group). metoprolol: β1 adrenergic receptor antagonist. The findings are shown as the mean ± SEM. Significant differences between treatment groups are marked with distinct letters (a and b) at a P-value of less than 0.05. [Time impact: F(3,40) = 2989.24, *P* < 0.05; treatment impact: F(2,40) = 2876.89, *P* < 0.05; treatment × time interaction: F(6,80) = 28.77; *P* < 0.05].

Compared with glycine alone, metoprolol (24 nmol) in combination with glycine did not affect cumulative nutritional consumption significantly (*P* ≥ 0.05), suggesting that β1 adrenergic receptors are not critical for mediating the hypophagic effects induced via glycine administration (Fig. 4).

#### ICI 118,551 (β2 adrenergic receptor antagonist)

**Fig. 5:**
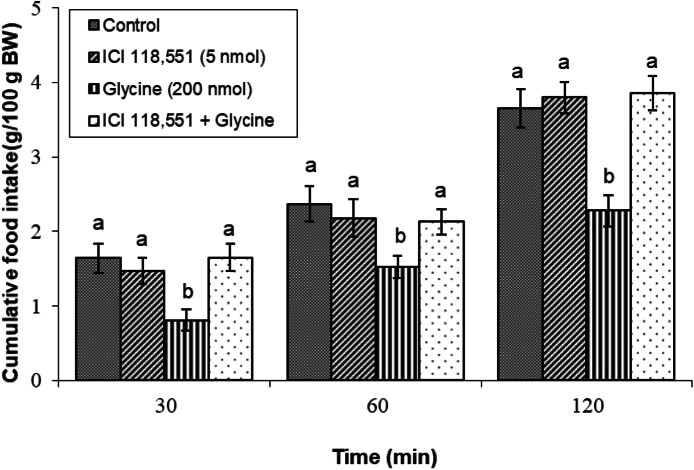
Changes in cumulative feed intake following ICV injection of ICI 118,551 (5 nmol), glycine (200 nmol) or their combination in young layer-type chickens (*n* = 11/group). ICI 118,551: β2 adrenergic receptor antagonist. The findings are shown as the mean ± SEM. Significant differences between treatment groups are marked with distinct letters (a and b) at a P-value of less than 0.05. [Time impact: F(3,40) = 3345.98, *P* < 0.05; treatment impact: F(2,40) = 2854.89, *P* < 0.05; treatment × time interaction: F(6,80) = 30.19; *P* < 0.05].

Coadministration of ICI 118,551 (5 nmol) with glycine resulted in significant inhibition of glycine-induced hypophagia, as indicated by a marked increase in cumulative feed consumption compared with the glycine-only treatment (*P* < 0.05). This result highlights the essential role of β2 adrenergic receptors in mediating the hypophagic effects associated with glycine administration (Fig. 5).

#### SR 59230R (β3 adrenergic receptor antagonist)

**Fig. 6:**
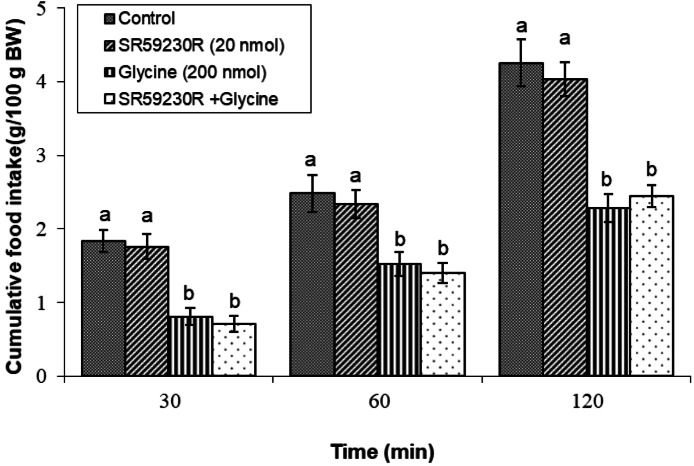
Changes in cumulative feed intake following ICV injection of SR 59230R (20 nmol), glycine (200 nmol) or their combination in young layer-type chickens (*n* = 11/group). SR 59230R: β3 adrenergic receptor antagonist. The findings are shown as the mean ± SEM. Significant differences between treatment groups are marked with distinct letters (a and b) at a P-value of less than 0.05. [Time impact: F(3,40) = 2761.89, *P* < 0.05; treatment impact: F(2,40) = 3124.82, *P* < 0.05; treatment × time interaction: F(6,80) = 26.83; *P* < 0.05].

Coadministration of SR 59230R (20 nmol) with glycine did not lead to any significant changes in cumulative nutritional intake relative to glycine-only injection (*P* ≥ 0.05), indicating that β3 adrenergic receptors do not significantly influence the hypophagic response induced by glycine (Fig. 6).

## Discussion

Glycine, a nonessential amino acid, has garnered attention in the field of nutritional neuroscience because of its role in regulating feeding behavior (He and Wu, 2020). Recent studies have highlighted its hypophagic effects, particularly in avian species such as broiler chickens (Rahimi et al., 2021a). Understanding glycine mechanisms and interactions with various neurotransmitter systems, including the adrenergic system, is crucial for developing strategies to optimize feeding practices in poultry.

Therefore, in our experiments, we examined the effects of glycine on meal consumption. Our results demonstrate that glycine administration (100-200 nmol ICV) significantly reduces food intake in layer-type chickens (*P* < 0.05), with effects persisting for 120 min post-injection. This effect aligns with glycine's role as an inhibitory neurotransmitter in the CNS, where it binds to glycine receptors, leading to neuronal hyperpolarization and an increased threshold for neuronal stimulation (Cioffi, 2021). Glycine interacts with DA pathways, particularly via D1 receptors, which are crucial for regulating appetite and reward mechanisms (Rahimi et al., 2021a). Previous research has shown that glycine influences feeding behavior by interacting with glutamatergic signaling pathways, adding complexity to the regulatory mechanisms governing appetite (Raiteri, 2024). Additionally, glycine receptors modulate feed consumption influenced by DA in broilers (Rahimi et al., 2021b). Recently, a study in broiler chickens demonstrated that central infusion of glycine at doses of 100 and 200 nmol significantly reduced meal consumption, consistent with our observations in layer-type chickens (Rahimi et al., 2021b). This hypophagic effect of glycine is believed to result from its role in delaying starvation signal transmission. Furthermore, the study revealed that the infusion of strychnine, a glycine receptor antagonist, inhibited the hypophagic effects of glycine. Despite these differences, the hypophagic effects of glycine observed in birds highlight its potential as a species-specific modulator of feeding behavior, warranting further investigation into its mechanisms.

Transitioning to another critical system, the adrenergic system also plays a key role in regulating meal consumption, which was addressed in this study. NE plays a significant role in stimulating meal consumption in poultry and mammals. Numerous research studies have explored the function of adrenergic receptors in controlling food consumption in mammalian models; for example, one study shows that the α2 agonist clonidine mimics the inhibitory effects of NE on hypothalamic cells, whereas the α2 antagonist yohimbine reverses these effects in rats (Wellman et al., 1993). Additionally, it seems that NE modulates food intake through β1 and β2 receptors; ICV administration of salbutamol—a β2 agonist—reduces food intake, whereas antagonists of this receptor type can lead to increased consumption in rodents (Kanzler et al., 2011). In domestic birds, adrenergic receptors exhibit varied distributions and functional roles across different regions of the brain. Specifically, α2 adrenergic receptors are associated with increased food intake, whereas β2 and β3 receptors are linked to reduced consumption of both food and water (Mahdavi et al., 2024). It has been shown that ICV infusion of NE remarkably increases meal consumption in layer-type chickens within 30 minutes post-administration (Dennis, 2016). Similar findings have been reported in pigeons, where NE administration increases food consumption (Hagemann et al., 1998). Moreover, antagonists targeting β1 and β2 receptors have been shown to decrease both water and food consumption in chickens (Baghbanzadeh et al., 2015). In the current investigation involving layer-type chickens, the doses of adrenergic antagonists administered were below effective levels, resulting in no significant changes in food intake when these antagonists were injected alone.

In the context of this study, the administration of adrenergic antagonists alongside glycine was designed to investigate how adrenergic receptors contribute to the glycine-induced hypophagia. Notably, co-administration of parazosin (an α1 antagonist), yohimbine (an α2 antagonist), metoprolol (a β1 antagonist), or SR 59230R (a β3 antagonist) did not significantly alter food intake when combined with glycine. These findings suggest that α1, α2, β1, and β3 adrenergic receptors are unlikely to be pivotal in modulating the appetite-suppressing effects of glycine. Conversely, the β2 adrenergic receptor antagonist significantly suppressed glycine-induced hypophagia, indicating that β2 receptors are essential for mediating the effects of glycine on meal consumption. This finding aligns with those of previous research that identified β2 receptors as key players in appetite regulation. For example, research has shown that the activation of β2 receptors can increase energy expenditure and reduce food consumption (Hostrup and Onslev, 2022). Moreover, several studies on chickens have reported the role of β2 receptors in mediating the effects of ghrelin, leptin, opioids, and nociceptin/orphanin FQ on feeding behavior (Nayebzadeh et al., 2020; Zendehdel and Hassanpour, 2014; Zendehdel et al., 2020; 2017a). Unfortunately, limited studies have examined the interactions between the adrenergic and glycinergic systems. One study found that glycine induced catecholamine release 22 % less than acetylcholine. The ratio of norepinephrine to epinephrine also differed: acetylcholine favored NE release, while glycine favored E secretion (Yadid et al., 1992). Additionally, research on rats indicates that activating α1-adrenergic receptors and glycine binding sites on the NMDA receptor may partially mitigate age-related deficits in spatial learning, suggesting that glycine modulates NMDA activity and influences cognitive functions related to feeding behavior (Riekkinen et al., 1997). Furthermore, neuroanatomical investigations have demonstrated the concurrent presence of glycinergic and adrenergic receptors within multiple cerebral regions, with notable prevalence in the brainstem and hippocampus (Chhatar and Lal, 2021; Lynch et al., 2017). These results emphasize the interaction between glycine and adrenergic signaling pathways and their collective influence on feeding behavior and appetite regulation. This aligns with studies identifying β2 adrenergic receptors as key players in appetite regulation, suggesting a pathway through which glycine may exert its hypophagic effects.

## Conclusion

In summary, this study elucidates the role of glycine in inducing hypophagia in neonatal layer chickens. Also, these findings underscore the pivotal involvement of β2 adrenergic receptors in mediating the hypophagic effects of glycine. These results contribute significantly to our understanding of amino acid and neurotransmitter interactions in regulating feeding behavior and have implications for optimizing nutritional practices in poultry production. Future research should investigate the downstream signaling pathways activated by β2 adrenergic receptors following glycine administration, as well as potential interactions with other neurotransmitter systems known to regulate appetite. Additionally, examining potential sex-based differences in these pathways and exploring the long-term effects of glycine supplementation on growth performance and feed efficiency would provide valuable insights for practical applications in poultry nutrition and management.

## Declaration of generative AI and AI-assisted technologies in the writing process

During the preparation of this work the author(s) used Perplexity AI in order to identify and correct potential grammatical errors and improve the overall flow and readability of the manuscript. After using this tool, the author(s) reviewed and edited the content as needed and take(s) full responsibility for the content of the published article.

## Declaration of competing interest

The authors report no conflicts of interest.
